# Clinical Benefits of Arterial Spin-Labeling Magnetic Resonance Imaging for Primary Diffuse Large B-cell Lymphoma of the Central Nervous System Presenting With Lymphomatosis Cerebri: A Case Report

**DOI:** 10.7759/cureus.67577

**Published:** 2024-08-23

**Authors:** Akira Okazaki, Tomohiro Yamasaki, Eri Kataoka, Mayu Fujihiro, Kazuhiko Kurozumi

**Affiliations:** 1 Department of Neurosurgery, Hamamatsu University School of Medicine, Hamamatsu, JPN; 2 Department of Diagnostic Pathology, Hamamatsu University School of Medicine, Hamamatsu, JPN

**Keywords:** cell density, perfusion imaging, large b-cell, lymphoma, central nervous system

## Abstract

Of the primary central nervous system (CNS) lymphomas, diffuse large B-cell lymphoma of the CNS (CNS-DLBCL) is an aggressive extranodal lymphoma that originates in the CNS. Lymphomatosis cerebri (LC) is an exceptionally rare subtype, posing diagnostic challenges due to the absence of abnormal enhancement and making the identification of suitable biopsy sites difficult. Arterial spin-labeling magnetic resonance imaging (ASL-MRI) is a non-invasive MRI technique that quantifies tumor blood flow. This report presents a case of CNS-DLBCL with LC, which was evaluated and biopsied using ASL-MRI of the brain. Herein, we present a case of a 32-year-old female who presented with abnormal involuntary movements and cognitive impairments. She underwent an MRI which showed a diffuse and infiltrative lesion in the bilateral basal ganglia, showing a high signal intensity area on fluid-attenuated inversion recovery (FLAIR) images with no contrast enhancement. Computed Tomography scans and Gallium-67 scintigraphy showed no abnormal uptake throughout the whole body. Although she received corticosteroid treatments, subsequent MRI showed an enlarged lesion, and she underwent a brain biopsy. The biopsy site was determined based on high perfusion demonstrated by ASL-MRI and the histological findings positive for B-cell markers led to diagnoses of CNS-DLBCL, specifically LC. Her symptoms improved following high-dose methotrexate and whole-brain irradiation. Subsequent MRI scans showed a dramatic improvement, and the high perfusion observed in the ASL-MRI disappeared. This report has emphasized the critical role of histopathology in diagnosing CNS-DLBCL presenting with LC, a highly aggressive lymphoma requiring prompt treatment. In this case, high ASL-MRI signal intensity indicated an increased area of tumor cell density suitable for biopsy. This is the first report to establish a relationship between cell density and ASL-MRI signal intensity in LC. The challenge in locating the optimal biopsy site due to the lack of contrast enhancement and the difference in tumor cell densities within high signal intensity areas on FLAIR imaging is presented. ASL-MRI provides information on tumor blood flow (TBF), which may be associated with higher tumor cell density, making it a valuable tool for identifying suitable biopsy sites. Thus, ASL-MRI is clinically beneficial for the biopsy of LC cases that show high signal intensity on FLAIR images without contrast enhancement.

## Introduction

Primary diffuse large B-cell lymphoma of the central nervous system (CNS-DLBCL), which accounts for 95% of primary CNS lymphomas, formerly known as primary CNS lymphoma (PCNSL). It is a rare and aggressive disease that accounts for approximately 2-4% of all primary brain tumors, and 4-6% of all extranodal lymphomas [1]. Magnetic resonance imaging (MRI) shows single or multiple lesions with varying degrees of mass effect and strong homogeneous or ring contrast enhancement. Lymphomatosis cerebri (LC) is an exceptionally rare subtype of CNS-DLBCL [2,3], making it challenging to diagnose due to its nonspecific clinical and neuroimaging features. This complexity necessitates a broad differential diagnosis, which can inadvertently lead to treatment delays [4]. The imaging features of LC include a diffusely infiltrating form of CNS-DLBCL with the absence of abnormal enhancement and mass-like lesions [2]. This lack of abnormal or mass-like enhancement of lesions on the initial MRI can result in false-negative biopsy results, because active tumor areas and vasogenic edema, which appear hyperintense on T2-weighted imaging (T2WI) and fluid-attenuated inversion recovery (FLAIR) images, cannot effectively be differentiated by computed tomography or conventional MRI [5,6].

Arterial spin-labeling MRI (ASL-MRI) is an MRI technique for perfusion measurement. This technique uses arterial blood water as a freely diffusible intrinsic tracer, making it a completely non-invasive method that does not require the administration of an intravenous contrast agent [7,8]. ASL-MRI has demonstrated clinical utility for differential diagnoses and for evaluating therapeutic effectiveness in brain tumors, based on the quantification of tumor blood flow (TBF) [9]. This report presents a case of CNS-DLBCL with LC, which was evaluated and biopsied using ASL-MRI of the brain. We also provide an overview of the patients’ clinical progress, supplemented with radiological and histopathological observations.

## Case presentation

**Figure 1:**
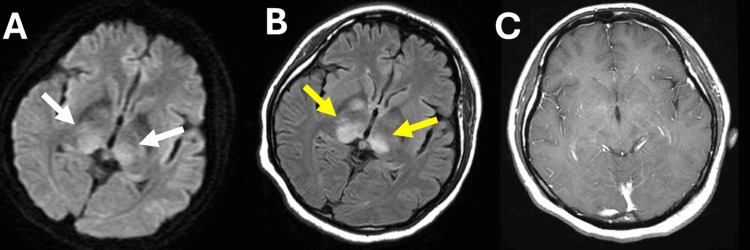
Initial magnetic resonance imaging findings. A) A diffusion-weighted imaging sequence revealed high-signal intensity areas (white arrows) in bilateral basal ganglia. B) A fluid-attenuated inversion recovery image revealed high-signal intensity areas (yellow arrows) in bilateral basal ganglia. C) No contrast enhancement was observed on a gadolinium T1-weighted image.

A 32-year-old immunocompetent woman initially presented to a hospital with hyperkinetic abnormal involuntary movement in her extremities, progressive cognitive impairment, and truncal ataxia, after experiencing overeating and weight gain for a period of 6 months. Upon admission, her blood pressure was 123/74 mmHg, heart rate was 110 beats/min without arrhythmia, and body temperature was 38.6°C. A neurological examination confirmed disorientation, and hyperkinetic abnormal involuntary movement in her extremities similar to ballism, and she was bedridden as a result of truncal ataxia. On MRI, diffusion-weighted images (DWI) (Figure 1A) and FLAIR images (Figure 1B) showed high-signal intensity areas in the bilateral basal ganglia, but no contrast enhancement was observed (Figure 1C).

**Figure 2:**
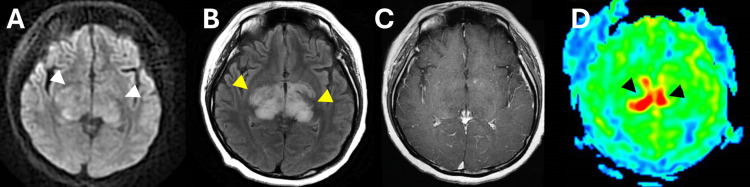
Magnetic resonance imaging (MRI) findings four months after the initial MRI. A) A diffusion-weighted imaging sequence revealed enlarged high-signal intensity areas (white arrowheads) in bilateral basal ganglia. B) A fluid-attenuated inversion recovery (FLAIR) image revealed enlarged high-signal intensity areas (yellow arrowheads) in bilateral basal ganglia. C) No contrast enhancement was observed on a gadolinium T1-weighted image. D) The arterial spin-labeling MRI revealed increased cerebral blood flow in narrower bilateral basal ganglia areas (black arrowheads) compared to the FLAIR image.

Blood tests showed that some tumor markers, including soluble interleukin 2 receptor (sIL-R2), were within the normal range and anti-nuclear antibodies were negative. Cerebrospinal fluid (CSF) levels of protein, sugar, and sIL-R2 were within the normal ranges, with no elevated cell counts or atypical cells. CT scans showed no abnormal findings, and Gallium-67 scintigraphy shows no abnormal uptake throughout the whole body. She received high-dose steroid treatment for the diagnosis of an inflammatory disease. As a result, her symptoms of consciousness disturbance and ballism-like movement improved temporarily, and the lesion showed slight shrinkage on MRI. However, her ballism-like movements reappeared, and she showed somnolence, with the expansion of the non-enhanced lesion on MRI four months after the initial MRI. She was referred to our hospital for histological evaluation of a brain biopsy. Subsequent MRI showed an enlarged DWI (Figure 2A) and FLAIR (Figure 2B) high-signal intensity area with no contrast-enhanced lesions (Figure 2C). A further MRI was performed to determine the exact location of the biopsy. ASL-MRI displayed increased cerebral blood flow in the bilateral midbrain to the thalamus (Figure 2D) whereas gadolinium T1-weighted images (Gd-T1WI) showed no contrast enhancement. H1-magnetic resonance spectroscopy demonstrated elevated lipid and lactate peaks combined with a high choline/creatine and choline/N-acetyl aspartate ratio, indicating a brain tumor.

**Figure 3:**
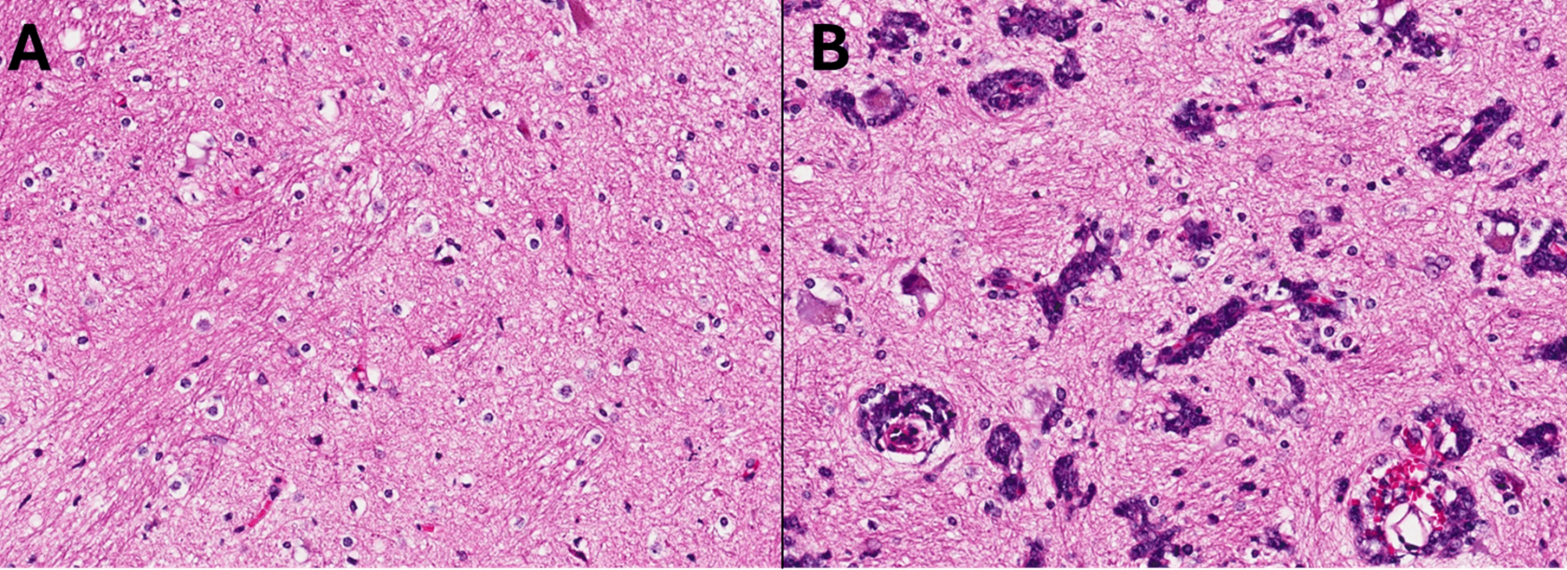
Differences in cell densities within high signal intensity areas on fluid-attenuated inversion recovery (FLAIR) images and the association of high signal intensity on arterial spin-labeling magnetic resonance imaging (ASL-MRI) with high tumor cell density. A) The hematoxylin and eosin (H&E) stained section from the initially sampled area, which showed high signal intensity on FLAIR images without high-perfusion areas on ASL-MRI, revealed edematous changes and a few scattered tumor cells around the blood vessels (magnification, ×200). B) The H&E-stained section from the area that showed high signal intensity on both FLAIR images and ASL-MRI exhibited the perivascular spread of large, round atypical cells with prominent nucleoli and mitotic figures, and high cell density (magnification, ×200).

She underwent stereotactic biopsy using the Leksell stereotactic system (Elekta KK, Tokyo) and Leksell SurgiPlan (Elekta KK, Tokyo) was used for determining the target sites. The initial target sites within a high signal intensity area on FLAIR images, away from the high-perfusion sites on ASL-MRI, were sampled. Since the intraoperative frozen section did not clearly identify tumor cells, we proceeded to the deeper high-perfusion area on ASL-MRI in the vicinity of the left nucleus ruber and subthalamic nucleus and sampled the sites, where the proliferation of tumor cells was confirmed. The microscopic images of the hematoxylin and eosin-stained sections from the initially sampled area, which showed a high signal intensity on FLAIR images without high-perfusion area on ASL-MRI, revealed edematous changes and a few scattered tumor cells around the blood vessels (Figure 3A). On the other hand, the specimen from the area that showed high signal intensity on both FLAIR images and ASL-MRI exhibited the perivascular spread of large, round atypical cells with prominent nucleoli and mitotic figures, and a high cell density (Figure 3B).

**Figure 4:**
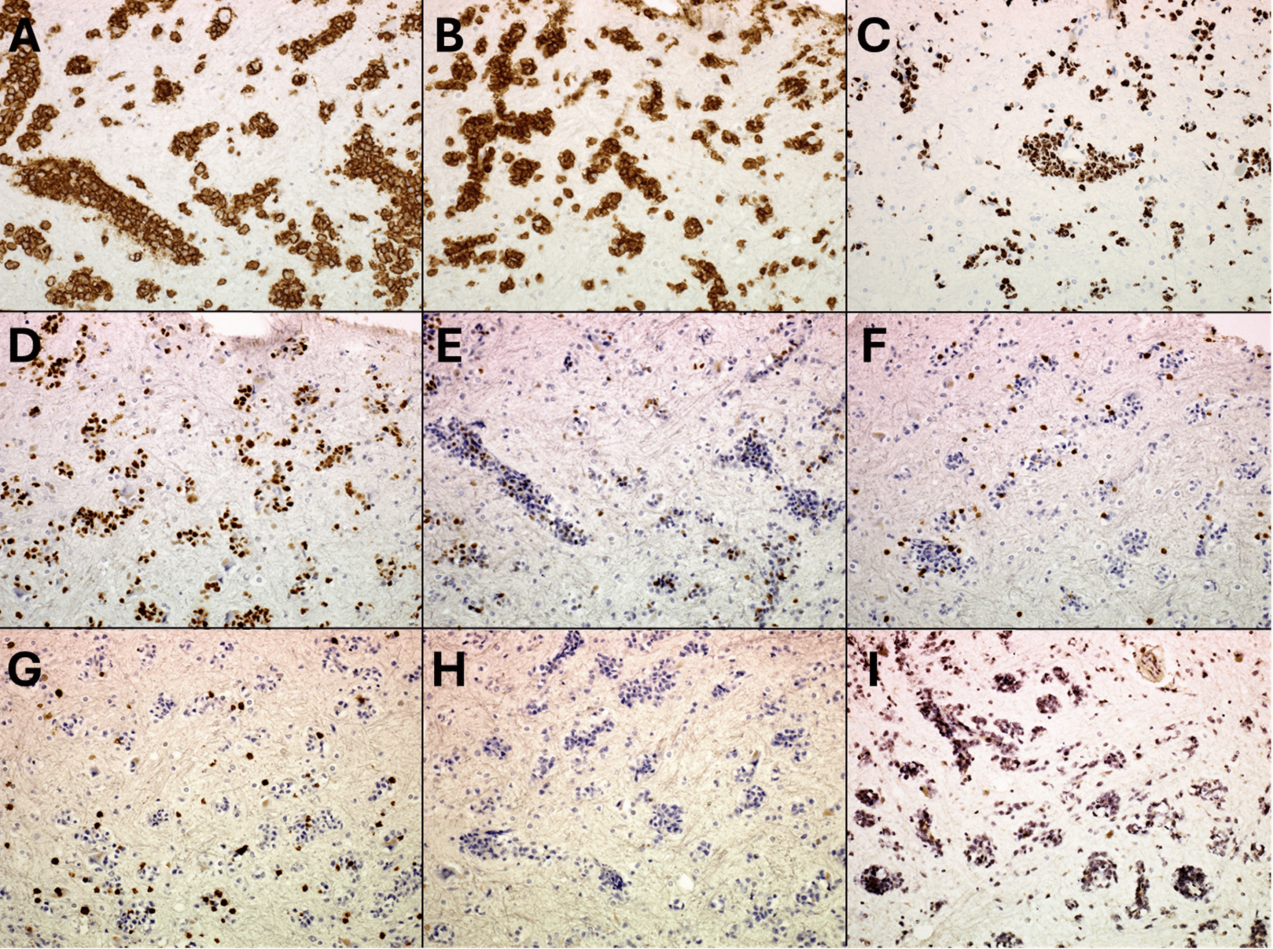
Pathological features of lymphomatosis cerebri in photomicrographs of the histopathology of the brain biopsy. On immunohistochemical staining, atypical lymphoid cells were strongly stained for the B-cell marker A) CD20 (magnification, ×200) and B) CD79a (magnification, ×200). C) The Ki-67 proliferation index was markedly increased in 50% of the tumor cells (magnification, ×200). D) Most of the tumor cells showed positive staining for Bcl6 (magnification, ×200) and E) partial positive staining for MUM1 (magnification, ×200), while negative staining was observed for F) CD3 (magnification, ×200), G) CD5 (magnification, ×200), H) CD23 (magnification, ×200), and I) EBER-ISH (magnification, ×200).

Immunohistochemistry showed positivity for the B-cell markers CD20 (Figure 4A) and CD79a (Figure 4B). The Ki-67 proliferation index was markedly increased in 50% of the tumor cells (Figure 4C). Additionally, most of the tumor cells exhibited positive staining for Bcl6 (Figure 4D) and partial positive staining for MUM1 (Figure 4E), while they were negative for glial fibrillary acidic protein, Olig2, CD3 (Figure 4F), CD5 (Figure 4G), and CD23 (Figure 4H). In situ hybridization yielded negative results for Epstein-Barr virus (Figure 4I).

**Figure 5:**
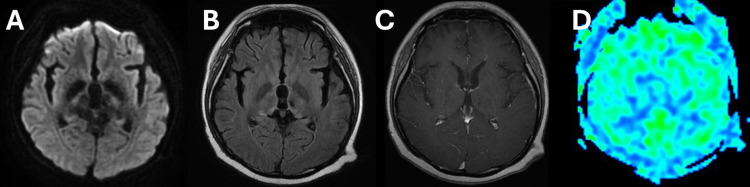
Post-treatment magnetic resonance imaging (MRI) findings. A) A diffusion-weighted imaging sequence and B) a fluid-attenuated inversion recovery image revealed that the high-signal intensity areas had dramatically shrunk with C) no contrast enhancement on a gadolinium T1-weighted image, and D) the high-perfusion sites on arterial spin-labeling MRI had diminished.

The diagnosis was CNS-DLBCL, specifically its infiltrative variant LC. On the basis of a diagnosis of CNS-DLBCL, the patient received three courses of high-dose methotrexate (3.5 g/m^2^) followed by whole-brain irradiation at a 30 Gy dose with a 14 Gy boost. The series of treatments was completed 3 months after the biopsy. Her symptoms of disturbed consciousness and abnormal involuntary movements in her extremities improved after treatment. The high-signal intensity areas in the bilateral midbrain to the bilateral basal ganglia region had shrunk on DWI (Figure 5A) and FLAIR (Figure 5B), and the high-perfusion sites on ASL-MRI had dramatically diminished (Figure 5C) with no contrast enhancement on Gd-T1WI (Figure 5D).

## Discussion

In this report, we presented a very rare case of LC. LC is a subtype of CNS-DLBCL and shows diffuse FLAIR high-signal lesions on initial MRI without clear enhancement or mass formation, often leading to delayed diagnosis [10]. Additionally, the lack of clearly enhanced masses makes it challenging to determine the target for biopsy [4]. In the present case, we successfully excised the lesions and confirmed the diagnosis of DLBCL by targeting lesions with high perfusion of ASL-MRI, allowing for treatment interventions. We presented the imaging and pathological findings, along with the treatment course of this rare case.

Bakshi et al. termed the rare form of diffusely infiltrating CNS-DLBCL, which lacks well-defined tumor masses, as LC in a case report published in 1999 [2]. LC is a rare variant of CNS-DLBCL, and the demographic characteristics of patients with LC closely resemble those with CNS-DLBCL. Both CNS-DLBCL and LC typically occur in individuals between the ages of 50 and 70 years, with similar distributions in males and females of 1.5:1 for CNS-DLBCL and 1.2:1 for LC. LC represents a diagnostic challenge, with only approximately one-third of patients receiving an accurate diagnosis during their lifetime [4]. A definitive pathological diagnosis of LC tends to take significantly longer (138 days) than that for CNS-DLBCL (generally approximately 70 days) in immunocompetent patients [11]. There are several possible reasons for this diagnostic delay. First, the clinical symptoms of LC can often be masked by cognitive changes or personality shifts, which can easily be mistaken for conditions like dementia or depression. In such cases, the immediate need for neuroimaging is often not recognized in standard medical practice [12]. Second, LC typically presents with similar neuroimaging findings to not only gliomatosis cerebri [6], but also CNS infections and inflammatory, toxic, and metabolic disorders on brain MRI [12,13], thus complicating the LC diagnosis. The diagnosis may also be delayed by the administration of corticosteroids prior to biopsy. Lymphoma cells are highly sensitive to corticosteroids, which can induce cell arrest, apoptosis, and transient tumor mass shrinkage via the p38-mitogen-activated protein kinase pathway, potentially further complicating a histopathological diagnosis. Ideally, corticosteroids should be avoided before biopsy, but may often be administered when patients present with neurological symptoms before consulting a neurooncological center [14]. The delay in diagnosis of LC has a negative influence on the timing of treatment decisions, which is associated with a poor prognosis. In the current cases, definitive biopsy-based pathological diagnoses of LC were made on days 161 after the initial visit in this case.

The diagnosis of LC is established on the basis of the following criteria: 1) the presence of diffuse and asymmetric T2 hyperintensity lesions impacting the cerebral white matter in at least three cerebral lobes or three anatomical areas of the CNS, 2) lesions show no enhancement or non-mass-like enhancement on initial MRI, and 3) the lesions are pathologically verified as CNS-DLBCL. Patients with systemic and intravascular lymphoma, as revealed by imaging studies or biopsies of lymph nodes, skin, bone marrow, or other suspicious lesions, are excluded [6,10,15]. Contrast-enhanced patterns of LC on MRI include non-enhancement, and non-mass-like enhancement on the initial MRI (patchy contrast enhancement is also allowed), as well as nodular or mass-like enhancement on later follow-up MRI [3]. The current case revealed diffuse, bilateral, and asymmetric T2 hyperintensity white matter lesions in more than three anatomical regions and were diagnosed pathologically with diffuse large B-cell lymphoma. No contrast enhancement was consistently observed in this case. Based on these findings, this case was diagnosed as LC.

Tumor diagnosis depends on the accurate identification of the target area for biopsy; however, tumor heterogeneity and the inability to identify the most malignant areas due to a lack of abnormal enhancement or no mass-like enhancement of the lesion on MRI can reduce this accuracy [16]. FLAIR imaging may be used to guide biopsies in non-enhancing tumors, but the high-intensity area on FLAIR imaging includes vasogenic edema and tumor infiltrative edema, which may not be tumor-specific findings, thus obscuring the precise tumor location [17].

In the present case, ASL-MRI was useful for determining the biopsy site. ASL-MRI is an MRI technique that uses arterial blood water as a tracer to measure blood flow in the brain non-invasively [18]. This technique is particularly useful for assessing perfusion in various clinical lesions, including stroke, neurodegenerative diseases, and brain tumors [4,19]. TBF measured by ASL-MRI was also reported to distinguish between high-grade and low-grade gliomas [9]. The intensity of the ASL-MRI signal correlates with vessel density and the MIB1 index, which are indicators of cell proliferation, thus aiding the diagnosis and treatment evaluation of brain tumors based on TBF measured by ASL-MRI. The increase in cell density creates a low-oxygen environment, leading to activation of hypoxia-inducible factor 1-alpha, which triggers the expression of vascular endothelial growth factor, promoting the formation of new blood vessels [20]. High ASL-MRI signal intensity may thus indicate an increased area of tumor cell density suitable for biopsy. In this case, the high signal intensity sites on FLAIR images without the high-perfusion area on ASL-MRI showed a few scattered tumor cells. On the other hand, the specimen from the area that showed high signal intensity on both FLAIR images and ASL-MRI exhibited high tumor cell density. This suggests that cell densities vary within high signal intensity areas on FLAIR images, and high signal intensity on ASL-MRI is associated with high tumor cell density. Thus, ASL-MRI is clinically beneficial for the biopsy of LC cases that show high signal intensity on FLAIR images without contrast enhancement.

## Conclusions

This report has highlighted the importance of histopathology in confirming the diagnosis of CNS-DLBCL presenting with LC, an aggressive type of lymphoma that requires rapid treatment. We presented the details of the case of LC in which ASL-MRI was useful for determining the biopsy site. To the best of our knowledge, this provides the first report of the relationship between tumor cell density and ASL-MRI signal intensity in LC. The lack of contrast enhancement and variable cell density within areas of high signal intensity on FLAIR images can make it difficult to locate the optimal site for biopsy in patients with LC. ASL-MRI can provide information on TBF, which may correlate with areas of higher cell density, and a high signal observed with ASL-MRI may thus serve as a valuable indicator for identifying the most suitable biopsy site. Thus, ASL-MRI is clinically feasible for the biopsy of LC cases that show high signal intensity on FLAIR images without contrast enhancement.

## References

[1] Kang MK, Ahn SJ, Ha J, Park SH, Moon J, Chu K (2023). Natural killer T-cell primary CNS lymphoma presenting as lymphomatosis cerebri: A case report and literature review. J Neuropathol Exp Neurol.

[2] Bakshi R, Mazziotta JC, Mischel PS, Jahan R, Seligson DB, Vinters HV (1999). Lymphomatosis cerebri presenting as a rapidly progressive dementia: Clinical, neuroimaging and pathologic findings. Dement Geriatr Cogn Disord.

[3] Fan M, Zhao L, Chen Q (2023). Clinical and imaging features of lymphomatosis cerebri: Analysis of 8 cases and systematic review of the literature. Clin Exp Med.

[4] Izquierdo C, Velasco R, Vidal N (2016). Lymphomatosis cerebri: A rare form of primary central nervous system lymphoma. Analysis of 7 cases and systematic review of the literature. Neuro Oncol.

[5] Rathore S, Akbari H, Doshi J (2018). Radiomic signature of infiltration in peritumoral edema predicts subsequent recurrence in glioblastoma: Implications for personalized radiotherapy planning. J Med Imaging.

[6] Li L, Rong JH, Feng J (2018). Neuroradiological features of lymphomatosis cerebri: A systematic review of the English literature with a new case report. Oncol Lett.

[7] Furtner J, Bender B, Braun C, Schittenhelm J, Skardelly M, Ernemann U, Bisdas S (2014). Prognostic value of blood flow measurements using arterial spin labeling in gliomas. PLoS One.

[8] Petcharunpaisan S, Ramalho J, Castillo M (2010). Arterial spin labeling in neuroimaging. World J Radiol.

[9] Noguchi T, Yoshiura T, Hiwatashi A (2008). Perfusion imaging of brain tumors using arterial spin-labeling: correlation with histopathologic vascular density. AJNR Am J Neuroradiol.

[10] Ruan Z, Chu L, Liu C, Hu Y, Huang J (2021). Lymphomatosis cerebri: Multimodality imaging features and misdiagnosis analysis. Oncol Lett.

[11] Haldorsen IS, Espeland A, Larsen JL, Mella O (2005). Diagnostic delay in primary central nervous system lymphoma. Acta Oncol.

[12] Lewerenz J, Ding XQ, Matschke J (2009). Dementia and leukoencephalopathy due to lymphomatosis cerebri. BMJ Case Rep.

[13] Kitai R, Hashimoto N, Yamate K (2012). Lymphomatosis cerebri: Clinical characteristics, neuroimaging, and pathological findings. Brain Tumor Pathol.

[14] Scheichel F, Marhold F, Pinggera D (2021). Influence of preoperative corticosteroid treatment on rate of diagnostic surgeries in primary central nervous system lymphoma: A multicenter retrospective study. BMC Cancer.

[15] Yamada SM, Tomita Y, Takahashi M, Kawamoto M (2022). A case of lymphomatosis cerebri presenting with rapid progression of dementia: A literature review. NMC Case Rep J.

[16] Sangaletti S, Iannelli F, Zanardi F (2020). Intra-tumour heterogeneity of diffuse large B-cell lymphoma involves the induction of diversified stroma-tumour interfaces. EBioMedicine.

[17] Jin T, Ren Y, Zhang H, Xie Q, Yao Z, Feng X (2019). Application of MRS- and ASL-guided navigation for biopsy of intracranial tumors. Acta Radiol.

[18] Golay X, Hendrikse J, Lim TC (2004). Perfusion imaging using arterial spin labeling. Top Magn Reson Imaging.

[19] Lindner T, Bolar DS, Achten E (2023). Current state and guidance on arterial spin labeling perfusion MRI in clinical neuroimaging. Magn Reson Med.

[20] Fischer I, Gagner JP, Law M, Newcomb EW, Zagzag D (2005). Angiogenesis in gliomas: Biology and molecular pathophysiology. Brain Pathol.

